# Automated pupillometry is a predictor of outcome of stroke patients: an observational, prospective, cohort study

**DOI:** 10.1093/braincomms/fcaf079

**Published:** 2025-02-18

**Authors:** Irene Scala, Massimo Miccoli, Jacopo Di Giovanni, Fabiana Cerulli, Pier A Rizzo, Simone Bellavia, Francesca Vitali, Francesca Colò, Serena Abruzzese, Giacomo della Marca, Valeria Guglielmi, Valerio Brunetti, Riccardo Di Iorio, Aldobrando Broccolini, Paolo Profice, Paolo Calabresi, Mauro Monforte, Giovanni Frisullo

**Affiliations:** Catholic University of the Sacred Heart, 00168 Rome, Italy; Department of Neuroscience, Sense Organs, and Thorax, Fondazione Policlinico Universitario A. Gemelli IRCCS, 00168 Rome, Italy; Catholic University of the Sacred Heart, 00168 Rome, Italy; Catholic University of the Sacred Heart, 00168 Rome, Italy; Catholic University of the Sacred Heart, 00168 Rome, Italy; Catholic University of the Sacred Heart, 00168 Rome, Italy; Catholic University of the Sacred Heart, 00168 Rome, Italy; Catholic University of the Sacred Heart, 00168 Rome, Italy; Catholic University of the Sacred Heart, 00168 Rome, Italy; Catholic University of the Sacred Heart, 00168 Rome, Italy; Catholic University of the Sacred Heart, 00168 Rome, Italy; Department of Neuroscience, Sense Organs, and Thorax, Fondazione Policlinico Universitario A. Gemelli IRCCS, 00168 Rome, Italy; Department of Neuroscience, Sense Organs, and Thorax, Fondazione Policlinico Universitario A. Gemelli IRCCS, 00168 Rome, Italy; Catholic University of the Sacred Heart, 00168 Rome, Italy; Department of Neuroscience, Sense Organs, and Thorax, Fondazione Policlinico Universitario A. Gemelli IRCCS, 00168 Rome, Italy; Department of Neuroscience, Sense Organs, and Thorax, Fondazione Policlinico Universitario A. Gemelli IRCCS, 00168 Rome, Italy; Catholic University of the Sacred Heart, 00168 Rome, Italy; Department of Neuroscience, Sense Organs, and Thorax, Fondazione Policlinico Universitario A. Gemelli IRCCS, 00168 Rome, Italy; Department of Neuroscience, Sense Organs, and Thorax, Fondazione Policlinico Universitario A. Gemelli IRCCS, 00168 Rome, Italy; Catholic University of the Sacred Heart, 00168 Rome, Italy; Department of Neuroscience, Sense Organs, and Thorax, Fondazione Policlinico Universitario A. Gemelli IRCCS, 00168 Rome, Italy; Department of Neuroscience, Sense Organs, and Thorax, Fondazione Policlinico Universitario A. Gemelli IRCCS, 00168 Rome, Italy; Department of Neuroscience, Sense Organs, and Thorax, Fondazione Policlinico Universitario A. Gemelli IRCCS, 00168 Rome, Italy

**Keywords:** stroke outcome, pupillary light reflex, automated pupillometry, NeurOptics, dilation velocity

## Abstract

Automated pupillometry (AP) is a rapid, non-invasive tool to assess the pupillary light reflex, extensively used for monitoring patients with traumatic brain injury. In acute ischaemic stroke, quantitative tools to monitor neurological status and predict outcome are lacking. This study aims to evaluate the ability of AP to predict stroke outcome, defined through the modified Rankin Scale (mRS) scores. In this observational, cohort study, we enrolled adult patients with anterior circulation stroke admitted to the stroke unit of a comprehensive stroke centre between 2021 and 2024 who underwent AP evaluation within 72 h of stroke onset. Exclusion criteria were: intracranial hypertension, severe eye diseases, pathologies involving the autonomic nervous system and lack of 3-month follow-up data. The AP evaluation was repeated three consecutive times in each patient using the NPi-200® and mean parameters of the two eyes and those of the eye homolateral and contralateral to the ischaemic lesion were considered. Mann–Whitney *U*-test, *t*-test and *χ*^2^-test were used for univariate comparisons. Binary and ordinal multivariable logistic regression models were used for the adjusted analysis. The primary outcome measure was the dichotomization of the 3-month mRS of 0–2 versus 3–6. Secondary outcomes were the score on the 3-month mRS, 3-month dichotomization of mRS 0–3 versus 4–6, and 3-month and in-hospital death. Receiver operating characteristic curves (ROC) were computed to evaluate the prognostic ability of AP. Two-hundred and nine patients (123 men, median age 75 years) were included in the study. Among included patients, 11 (5.3%) died during the hospital stay and 124 (59.33%) had a 3-month mRS < 3. In multivariable logistic regression models corrected for all possible confounders, a low dilatation velocity (DV) in the eye homolateral to the stroke lesion was an independent predictor of poor prognosis, defined as both mRS > 2 and mRS > 3 at 3 months (*P* = 0.028 and *P* = 0.024, respectively). Furthermore, homolateral DV resulted to be a significant predictor of a shift towards a better outcome on the 3-month mRS in the ordinal logistic regression (*P* = 0.036). A DV ≥ 0.865 mm/s was able to predict a good stroke outcome at 90 days with 60% sensitivity and specificity (area under the curve 0.651; *P* < 0.001). No other AP parameters were independent predictors of stroke outcome. A reduction in the DV in the eye ipsilateral to the ischaemic lesion is associated with poor in-hospital and 3-month stroke outcome, and it could be useful for identifying patients who need a tailored monitoring and treatment path to improve their prognosis.

## Teaser

Scala *et al*. report that a reduction in the pupillary dilation velocity in the eye homolateral to the ischaemic lesion in an automated pupillometry assessment performed within 72 h of ischaemic stroke onset is predictive of poor outcome at 3 months, defined as a modified Rankin Scale >2.

## Graphical Abstract

**graphical abstract:**
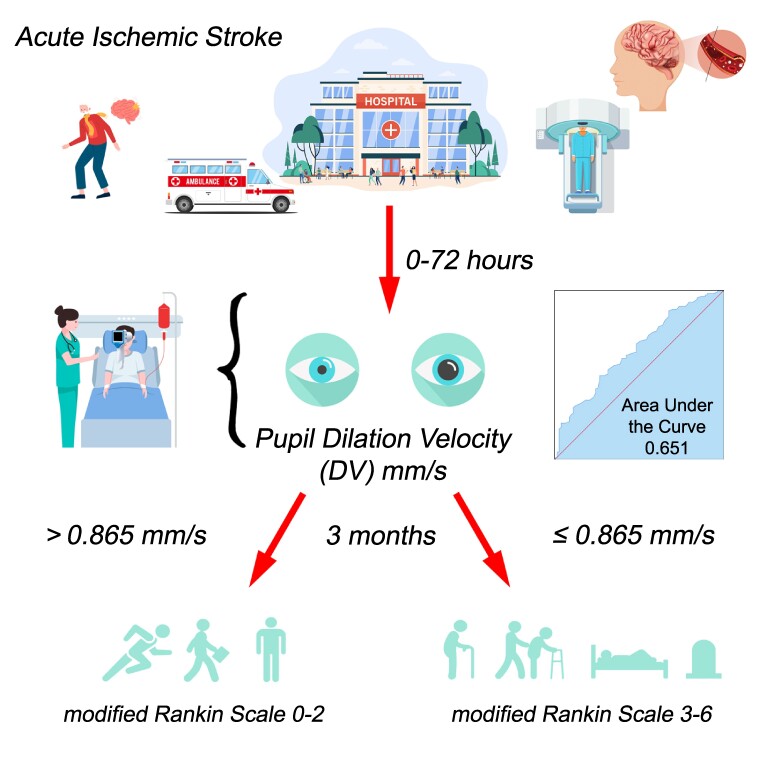

## Introduction

In the acute stroke setting, a non-invasive, quantitative, operator-independent tool for monitoring the neurological status is an unmet need. The pupillary reaction to light, namely the pupillary light reflex (PLR), is a subcortical reflex mediated by the autonomic nervous system that aims to achieve the best image resolution on the retina despite changes in external lighting.^1^ The role of PLR assessment is well established in intensive care units for the diagnostic and prognostic evaluation of neurologically injured, critically ill patients,^2^ since its alteration is an index of brainstem injury.^3^ Traditionally, PLR assessment has been performed qualitatively using a hand-held light source with a high inter-observer variability.^4^

To overcome these shortcomings, automated pupillometries (APs), devices capable of performing a PLR assessment in an operator- and external-lighting independent way,^5^ have started to gain traction in the context of intensive care units. Modern APs are able to decompose the PLR into several parameters and to compute an overall pupil function score, the neurological pupil index (NPi), a scalar value ranging from 0 to 5.^6^ Several studies have demonstrated the prognostic value of NPi in patients with acute intracranial pathologies^7^ due to its sensitivity to increased levels of intracranial pressure.^8^ However, this value seems less sensitive in identifying cortical damage not associated with intracranial hypertension compared with other pupillary parameters.^9^

Among pupillary parameters recorded by APs, pupillary dilatation velocity (DV) appears to be the most influenced by cortical, descending inputs to the brainstem, since pupillary dilation is strongly influenced by cognitive tasks and emotional stimuli.^10^ Previous studies have already demonstrated a close correlation between DV and level of consciousness in patients with brain injury^11^ and a reduction in this parameter was associated with delirium in patients with acute ischaemic stroke.^12^ Furthermore, ischaemic lesions in strategic cortical areas were associated with mild alterations in pupillary DV.^13^

Although the prognostic role of AP has been widely explored in critically ill patients with acquired brain injury,^7,14^ less is known about its possible utilization in patients with acute ischaemic stroke, which is one of the leading causes of mortality and disability worldwide.^15-17^ Published studies have been conducted on heterogeneous populations of patients admitted to intensive care units with acute intracranial pathologies, including acute ischaemic stroke, and have reported promising results regarding the ability of NPi to predict short-term^18,19^ and long-term^20^ outcomes. However, no data are available on the prognostic ability of APs in patients with non-critical acute ischaemic stroke who do not experience intracranial hypertension during the acute phase of the disease, for whom NPi may not be the most appropriate AP parameter for identifying cortical damage.^9^

Based on these premises, the aim of our study was to evaluate the ability of an early complete AP assessment (within 72 h of stroke onset), and, in particular, of pupillary DV, to predict 3-month and in-hospital stroke outcome.

## Materials and methods

### Study design and population

In this single-centre, observational, prospective, cohort study, we included a convenience sample of adult (≥18 years) patients admitted to the stroke unit of Fondazione Policlinico Universitario A. Gemelli IRCCS from March 2021 to January 2024 for a primary diagnosis of anterior circulation acute ischaemic stroke, who underwent AP assessment within 72 h of stroke onset. We chose the 72-h cut-off since it is widely recognized as the acute phase of stroke, both for the high risk of stroke recurrence, the occurrence of brain swelling and haemorrhagic transformations, that are most likely to occur during this timeframe.^21^ Patients were excluded if they met one of the following: discharge diagnosis other than ischaemic stroke; medical history of previous eye surgery, major eye trauma or major eye diseases (i.e. glaucoma, cataracts requiring surgical intervention, severe retinopathy and previous optic neuritis); midline shift >5 mm at baseline or follow-up neuroradiological examination; history of previous neurological diseases affecting the autonomic nervous system (i.e. Parkinson disease, multiple system atrophy, other parkinsonism and previous diagnosis of autonomic neuropathy); posterior circulation stroke and missing data about the 3-month follow-up.

The extended list of the abbreviations cited in this article is available in Supplementary Table 1. The study was conducted according to the Strengthening the Reporting of Observational Studies in Epidemiology reporting (STROBE) guidelines. This study complied with the principles of the 1964 Declaration of Helsinki and its later amendments. The research protocol was approved by the Institutional Review Board—Comitato Etico of Fondazione Policlinico Universitario ‘A Gemelli’ IRCCS—Rome (Prot. n°0031530/22). All study participants or, in case of diminished capacity, their legal representatives gave their written informed consent to participate in the study.

### Automated pupillometry

Six trained investigators (I.S., P.A.R., F.C., M.M., J.D.G. and F.V.) performed the AP assessment in both eyes of all study subjects using NPi-200® (NeurOptics, Irvine, CA, USA). To minimize interference from external lighting and circadian rhythm influence, all automated PLR evaluations were performed between 6:00 and 8:00 p.m., while the patient was lying on an SU bed. The PLR assessments were repeated three consecutive times for each patient, considering for the analysis the mean value of the three measurements for the subsequent statistical analyses for each parameter. The three consecutive pupillary assessments were performed 30–60 s apart, considering the time needed by the machine to perform a measurement. We first evaluated the eye contralateral to the ischaemic lesion. All data recorded by NPi-200® were then reported in an electronic case report form. The mean AP values of the two eyes and the AP parameters of the eye homolateral and contralateral to the ischaemic lesion were considered for further analysis (excluding patients with bilateral strokes, for whom only the mean values were considered). Furthermore, we considered the absolute difference of the mean AP parameters of the two eyes. An extensive explanation of the parameters recorded by NPi-200® is provided in Supplementary Table 2.

### Clinical and demographic data collection

Clinical data were collected by three investigators experienced in stroke management (I.S., J.D.G. and F.C.) through a review of patients’ electronic medical records. The following variables were collected for each study participant: demographics (age, sex), baseline modified Rankin Scale (mRS) and the National Institute of Health Stroke Scale (NIHSS) at admission, stroke pathogenesis according to the Trial of Org 10172 in Acute Stroke Treatment (TOAST) classification, acute revascularization treatments for stroke, cardiovascular risk factors and other comorbidities, the assumption of concomitant pharmacological drugs able to impact the autonomic functioning at the time of the AP assessment (ACE inhibitors, sartans, α-blockers, β-blockers, SSRIs and calcium channel blockers). Smoking habit was defined as follows: A current smoker was defined as a patient who was smoking at the time of stroke onset or a patient who stopped smoking less than 6 months before stroke onset, a former smoker was defined as a patient who had previously smoked but who had stopped smoking more than 6 months before stroke onset, while a non-smoker was defined as a patient who had never smoked.^22^ Obesity was defined as a body mass index ≥30 kg/m^2^.

All patients underwent at least two neuroradiological examinations (brain CT/MRI), at the time of hospital admission and at 24–72 h follow-up. Some patients underwent more neuroradiological examinations according to clinical judgement. The neuroradiological characteristics of study patients were visually examined by two investigators (I.S. and G.F.) who visually analysed all available patients’ MRI/CT scans to obtain the following information: burden of white matter lesions according to periventricular white matter (PVWM) and deep white matter (DWM) Fazekas scales,^23^ haemorrhagic conversion of the ischaemic lesion, radiological evidence of intracranial hypertension (defined as a ≥5 mm deviation of the septum pellucidum from the ideal midline between the most anterior and posterior part of the falx cerebri^24^). The volume of the ischaemic lesions of included patients was independently calculated by both investigators according to the ABC/2 formula measured on DWI-weighted MRI scans^25^ performed 24–72 h after stroke unit admission or, when a brain MRI was not available, on a 24 h follow-up CT scan.^26^ The mean values of the volumes of the two raters were used in the analysis. The diagnosis of large vessel occlusion (LVO) was made using baseline cranial CT including CT-angiography.

### Outcome assessment

Clinical outcome was measured using the 3-month mRS score. The mRS scores were collected for each study participant by three expert investigators (I.S., S.B. and P.A.R.) during a routine follow-up outpatient visit or, alternatively, through a structured phone interview with the patient or a healthcare provider after 3 months ± 15 days from the stroke onset. All 3-month mRS assessors were blinded to the AP results at the time of the visit/telephone call. The primary outcome measure was a 3-month mRS of 0–2 (functional independence), since this dichotomization of the scale is usually used to define a ‘good outcome’.^27^ Secondary outcome measures were: the score on the 3-month mRS, 3-month mRS of 0–3 (independent ambulation), in-hospital death and 3-month death.

### Statistical analysis

The normality of data distribution was tested using the Shapiro–Wilk test. Numerical variables were summarized as median and interquartile range. Categorical variables are expressed as numbers and percentages. Univariate comparisons were performed using the Mann–Whitney *U*-test or unpaired *t*-test for numerical variables and by the *χ*^2^-test or Fisher’s exact test for categorical variables, as appropriate. In order to find independent predictors of study outcomes (3-month mRS 0–2 versus >2, 3-month mRS 0–3 versus >3), we ran multiple multivariable logistic regression models, including most of the variables that reached the statistical significance (*P* < 0.05) in the univariate comparison for the primary outcome measure and one selected by expert opinion^28^ (e.g. intravenous thrombolysis). All candidate variables were entered into binary logistic regression analyses, with a backward stepwise elimination approach set to simplify the model. The Hosmer–Lemeshow goodness-of-fit test was used to assess the calibration of the regression models. Given that only 11 patients died during hospitalization, and 28 after 3 months from stroke onset, we did not carry out a logistic regression model for these outcomes, as they would not have been reliable. A multivariable ordinal logistic regression was then computed for calculating the adjusted common odds ratio (OR) for a shift in the direction of a worse outcome on the 3-month mRS. The ordinal multivariable logistic regression model was computed with the same variables chosen for the binary logistic regression ones. The collinearity of the model was tested through the variance inflation factor (VIF). Missing data have not been imputed.

The results of the logistic regression models were expressed as ORs and 95% confidence intervals (CIs). Receiver operating characteristic (ROC) curves were carried out to evaluate the discriminating power of AP parameters in predicting the clinical outcome; optimal cut-offs for each AP variable were chosen based on the Youden index. Interrater reliability for the calculation of stroke volumes through the ABC/2 formula was evaluated with the intraclass correlation coefficient (ICC), using a two-way random effects model, single measures, absolute agreement. The ICC value was interpreted according to the guidelines suggested by Cicchetti (<0.40 = poor; 0.40–0.59 = fair; 0.60–0.74 = good; 0.75–1.00 = excellent).^29^ Statistical significance was set for two-tailed *P* < 0.05. Data were analysed using SPSS v26.0 (SPSS®, Inc., Chicago, IL, USA).

## Results

### Characteristics of the study population

**Figure 1:**
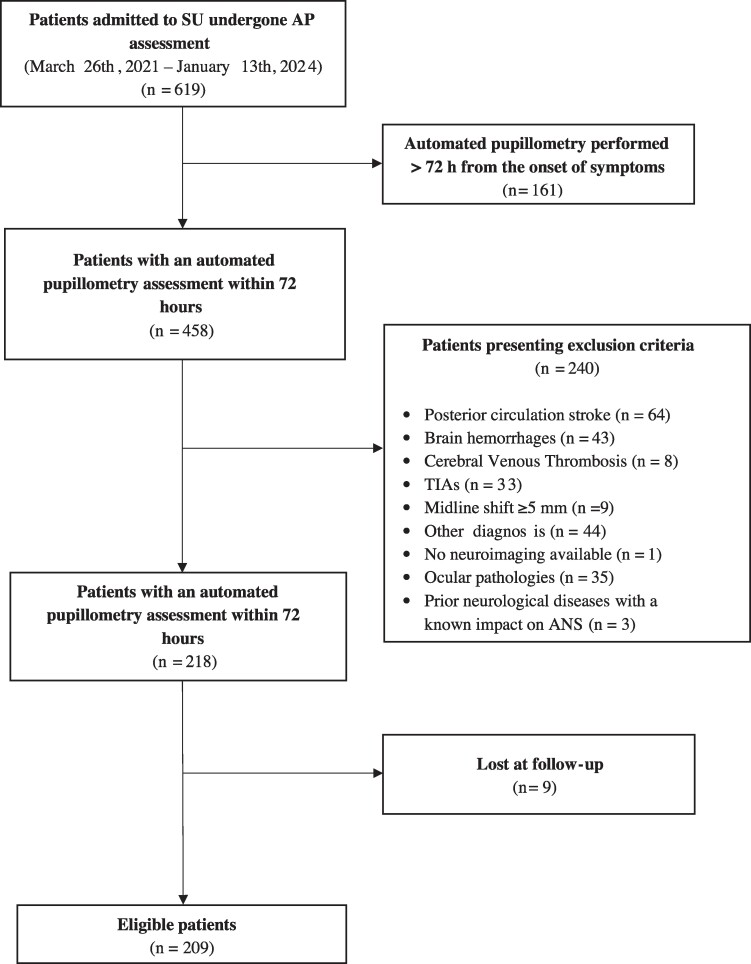
**The flow diagram of the study.** AP, automated pupillometry; SU, stroke init; h, hours; TIA, transient ischaemic attack; ANS, autonomic nervous system.

Between March 2021 and January 2024, 619 patients underwent AP assessment. In 161 patients, the pupillary evaluation was performed more than 72 h after the onset of symptoms. Two-hundred and forty of the remaining 458 presented at least an exclusion criterion. Finally, nine patients were lost to follow-up. The final analysis was then performed on 209 patients. For details, refer to the flow diagram of the study (Fig. 1).

The median age of the overall study population was 75.00 (63.50–82.00) years, and 123 (58.85%) were men. The mean time elapsed from the onset of symptoms and the AP assessment was 41.2 ± 23.3 h. Cardioembolism was the most represented pathogenic mechanism (63, 30.14%), followed by large artery atherosclerosis (41, 19.62%), while in about one-fourth of the study population, the pathogenetic mechanism was still unknown at the 3-month follow-up (51, 24.40%). None of the patients received any sedative agents or vasopressors within 24 h prior to the AP assessment. The distribution of the 3-month mRS of the study population was as follows: 50 patients (23.92%) had a mRS = 0, 41 (19.62%) a mRS = 1, 33 (15.79%) a mRS = 2, 19 (9.10%) a mRS = 3, 31 (14.83%) a mRS = 4, 7 (3.35%) a mRS = 5 and 28 patients (13.40%) died to follow-up. The median volume of the ischaemic lesion was 5.88 (0.93–26.06) cm^3^. The ICC value confirmed the excellent interrater agreement in the stroke volume evaluation (ICC = 0.936, 95% CI = 0.914–0.953).

A detailed description of the study population is provided in Table 1.

**Table 1:** The clinical and radiological characteristics of the whole study population and the univariate comparisons between patients with good (mRS 0–2) and poor (mRS > 2) 3-month clinical outcomes

		Whole population (*n* = 209)	Good outcome (mRS 0–2, *n* = 124)	Poor outcome (mRS 3–6, *n* = 85)	*P*-value
Demographic characteristics	Age (years)	75.0 (63.50–82.00)	71.00 (57.75–79.00)	79.00 (71.00–85.00)	**<0**.**001**
	Male sex	123 (58.85%)	84 (67.74%)	39 (45.88%)	**0**.**002**
Clinical features	Pre-admission mRS	0.00 (0.00–1.00)	0.00 (0.00–0.00)	1.00 (0.00–1.00)	**<0**.**001**
	Admission NIHSS	6.00 (2.00–13.50)	4.00 (2.00–9.00)	9.00 (5.00–20.00)	**<0**.**001**
Revascularization treatments	Intravenous thrombolysis	67 (32.06%)	40 (32.26%)	39 (45.88%)	0.113
	Endovascular treatment	79 (37.80%)	45 (36.29%)	22 (25.88%)	**0**.**046**
Radiological features	LVO	123 (58.85%)	60 (48.39%)	63 (75.00%)	**<0**.**001**
	Stroke volume (cm^3^)	5.88 (0.93–26.06)	2.58 (0.60–12.92)	17.69 (1.93–72.56)	**<0**.**001**
	DWM Fazekas	1.00 (0.00–2.00)	1.00 (0.00–1.00)	1.00 (1.00–2.00)	**0**.**001**
	PVWM Fazekas	1.00 (1.00–2.00)	1.00 (0.25–2.00)	2.00 (1.00–2.00)	**0**.**007**
	Haemorrhagic conversion	65 (31.10%)	28 (22.58%)	37 (43.53%)	**0**.**001**
TOAST classification	Large artery atherosclerosis	41 (19.62%)	18 (14.52%)	23 (27.06%)	0.093
	Cardioembolism	63 (30.14%)	35 (28.23%)	28 (32.94%)	
	Lacunar	38 (18.18%)	26 (29.97%)	12 (14.12%)	
	Other determined etiologies	16 (7.66%)	12 (9.68%)	4 (4.71%)	
	Cryptogenic	51 (24.40%)	33 (26.61%)	18 (21.18%)	
Comorbidities	Diabetes	50 (23.92%)	18 (14.52%)	32 (37.65%)	**<0**.**001**
	Hypertension	169 (80.86%)	95 (76.61%)	74 (87.06%)	0.059
	Dyslipidemia	94 (44.98%)	56 (46.16%)	38 (44.71%)	0.948
	Obesity	35 (16.75%)	18 (14.52%)	17 (20.0%)	0.297
	Former smokers	12 (5.74%)	8 (6.45%)	4 (4.71%)	0.413
	Current smokers	39 (18.67%)	26 (21.00%)	13 (15.29%)	0.293
	Atrial fibrillation	53 (25.36%)	26 (21.00%)	27 (31.76%)	0.078
	Cancer	33 (15.79%)	18 (14.52%)	15 (17.65%)	0.542
	Liver disease	5 (2.39%)	3 (2.42%)	2 (2.35%)	0.975
	Kidney failure	19 (9.09%)	11 (8.87%)	8 (1.18%)	0.894
Concomitant drugs	β-Blockers	96 (45.93%)	47 (37.90%)	49 (57.65%)	**0**.**005**
	α-Blockers	31 (14.83%)	21 (16.94%)	10 (11.76%)	0.302
	ACE inhibitors	106 (50.72%)	58 (46.77%)	48 (56.47%)	0.168
	Sartans	48 (22.97%)	31 (25.00%)	17 (20.00%)	0.399
	Calcium channel blockers	98 (46.89%)	41 (33.06%)	57 (67.06%)	0.315
	SSRIs	9 (4.31%)	4 (3.23%)	5 (5.88%)	0.353

mRS, modified Rankin Scale; NIHSS, National Institute of Health Stroke Scale; LVO, large vessel occlusion; DWM, deep white matter; PVWM, periventricular matter; ACE, angiotensin-converting enzyme; SSRIs, selective serotonin reuptake inhibitors. Significant findings are reported in bold.

### Univariate comparisons

Considering our primary outcome measure (3-month mRS 0–2 versus mRS 3–6), older age (*P* < 0.001), female sex (*P* = 0.002), higher NIHSS score at admission (*P* < 0.001) and a higher pre-morbid mRS (*P* < 0.001) were associated with a higher risk of functional dependence at 3 months. Patients who underwent mechanical thrombectomy had a better outcome than untreated patients (*P* = 0.046), while intravenous thrombolysis was not significantly different between groups. Diabetes (*P* = 0.015) was more frequent in patients with a poor outcome. Pharmacological treatments did not differ significantly between groups, except for β-blockers that were taken more frequently by the unfavourable outcome group (*P* = 0.005). Regarding the radiological characteristics of the stroke lesion, patients with a mRS > 2 at 3 months more frequently had a LVO (*P* < 0.001) and developed haemorrhagic conversion (*P* = 0.001) than the others. In addition, subjects with an unfavourable outcome had higher median PVWM and DWM Fazekas scores (*P* = 0.007 and *P* = 0.001, respectively) and stroke volumes (*P* < 0.001) than the others. Details are provided in Table 1.

Focusing on AP parameters, we can appreciate that a reduction in mean DV (*P* < 0.001), as well as a reduction of the same parameter in the eye homolateral (*P* < 0.001) and contralateral (*P* = 0.001) to the stroke lesion were strongly associated with an unfavourable outcome. Similar findings were obtained for the mean (*P* = 0.007), homolateral (*P* = 0.029) and contralateral (*P* = 0.006) average constriction velocity (CV), contralateral percentage of constriction (CH) (*P* = 0.026) and contralateral (*P* = 0.008) and mean maximum constriction velocity (MCV) (*P* = 0.007). No differences were detected in other AP parameters. Please refer to Table 2 for details.

**Table 2:** Automated pupillometry parameters of the entire study population and a comparison between the good (mRS 0–2) and poor (mRS > 2) 3-month outcome groups

Automated pupillometry	Whole population (*n* = 209)	Good outcome (mRS 0–2, *n* = 124)	Poor outcome (mRS 3–6, *n* = 85)	*P*
NPi mean	4.55 (4.30–4.70)	4.55 (4.35–4.72)	4.55 (4.27–4.70)	0.447
NPi homolateral	4.60 (4.40–4.70)	4.60 (4.40–4.72)	4.60 (4.30–4.70)	0.727
NPi contralateral	4.53 (4.30–4.70)	4.53 (4.30–4.70)	4.53 (4.30–4.70)	0.747
NPi absolute difference	0.10 (0.07–0.30)	0.10 (0.07–0.29)	0.10 (0.07–0.30)	0.692
BPD mean (mm)	3.30 (2.78–3.77)	3.37 (2.79–3.83)	3.17 (2.76–3.66)	0.144
BPD homolateral (mm)	3.29 (2.77–3.73)	3.37 (2.78–3.84)	3.21 (2.74–3.58)	0.165
BPD contralateral (mm)	3.29 (2.82–3.87)	3.40 (2.85–3.99)	3.18 (2.79–3.72)	0.089
BPD absolute difference (mm)	0.30 (0.14–0.54)	0.30 (0.10–0.52)	0.29 (0.15–0.57)	0.358
MIN mean (mm)	2.32 (2.00–2.61)	2.32 (2.01–2.64)	2.31 (1.97–2.52)	0.257
MIN homolateral (mm)	2.31 (1.97–2.59)	2.31 (2.00–2.63)	2.31 (1.92–2.55)	0.451
MIN contralateral (mm)	2.32 (1.99–2.64)	2.33 (2.01–2.65)	2.29 (1.97–2.58)	0.221
MIN absolute difference (mm)	0.16 (0.07–0.29)	0.16 (0.07–0.29)	0.18 (0.07–0.30)	0.411
CH mean (%)	30.00 (23.75–34.50)	30.25 (24.63–35.38)	30.0 (21.0–34.0)	0.089
CH homolateral (%)	30.00 (25.00–34.00)	30.0 (25.00–35.00)	30.0 (240–34.0)	0.367
CH contralateral (%)	30.00 (23.00–36.00)	31.00 (24.00–36.00)	29.00 (20.00–35.00)	**0**.**026**
CH absolute difference (%)	4.00 (2.00–6.50)	3.00 (2.00–6.00)	4.00 (2.00–8.00)	0.056
CV mean (mm/s)	1.98 (1.46–2.36)	2.01 (1.56–2.42)	1.89 (1.19–2.26)	**0**.**007**
CV homolateral (mm/s)	1.90 (1.48–2.36)	1.92 (1.54–2.44)	1.81 (1.33–2.26)	**0**.**029**
CV contralateral (mm/s)	1.97 (1.42–2.44)	2.07 (1.63–2.49)	1.86 (1.13–2.29)	**0**.**006**
CV absolute difference (mm/s)	0.30 (0.15–0.59)	0.30 (0.12–0.58)	0.33 (0.17–0.65)	0.365
MCV mean (mm/s)	2.92 (2.24–3.58)	3.00 (2.35–3.67)	2.84 (1.86–3.41)	**0**.**007**
MCV homolateral (mm/s)	2.86 (2.14–3.54)	2.86 (2.25–3.61)	22.80 (2.05–3.46)	0.079
MCV contralateral (mm/s)	2.90 (2.15–3.70)	3.01 (2.34–3.79)	2.74 (1.76–3.56)	**0**.**008**
MCV absolute difference (mm/s)	0.42 (0.14–0.77)	0.40 (0.12–0.79)	0.42 (0.17–0.75)	0.800
LAT mean (s)	0.240 (0.215–0.270)	0.235 (0.215–0.270)	0.250 (0.225–0.270)	0.232
LAT homolateral (s)	0.230 (0.200–0.270)	0.230 (0.200–0.270)	0.240 (0.230–0.270)	0.238
LAT contralateral (s)	0.230 (0.230–0.270)	0.230 (0.230–0.270)	0.240 (0.230–0.270)	0.216
LAT absolute difference (s)	0.030 (0.00–0.040)	0.030 (0.00–0.030)	0.030 (0.00–0.040)	0.436
DV mean (mm/s)	0.89 (0.72–1.07)	0.92 (0.78–1.15)	0.82 (0.62–0.96)	**<0**.**001**
DV homolateral (mm/s)	0.87 (0.69–1.08)	0.96 (0.74–1.15)	0.79 (0.62–1.01)	**<0**.**001**
DV contralateral (mm/s)	0.89 (0.68–1.07)	0.95 (0.75–1.11)	0.84 (0.62–1.00)	**0**.**001**
DV absolute difference (mm/s)	0.15 (0.07–0.23)	0.14 (0.07–0.26)	0.15 (0.63–0.23)	0.907

mRS, modified Rankin Scale; NPi, neurological pupil index; BPD, baseline pupil diameter; MIN, minimum pupil diameter; CH, percentage of constriction; CV, average constriction velocity; MCV, maximum constriction velocity; LAT, reflex LATency; DV, dilation velocity. Significant findings are reported in bold.

Similar results were found for the mRS 0–3 versus mRS > 3 comparison, both with regard to clinical and AP parameters. Data regarding clinical, radiological and AP comparisons between these groups are shown in Supplementary Tables 3 and 4, respectively. Similar results were also found for the comparison of mRS 0–5 versus mRS 6 after 3 months, both with regard to clinical and radiological features, except for atrial fibrillation and renal failure which were more frequent in deceased patients than the others (*P* = 0.022 and *P* = 0.002, respectively; Supplementary Table 5).

In contrast, in addition to all the DV and CV parameters, several other pupillary parameters differed between mRS 0–5 and mRS 6 groups, such as homolateral baseline pupil diameter (BPD) (*P* = 0.045), homolateral minimum pupil diameter (MIN) (*P* = 0.045), mean CH (*P* = 0.013) and homolateral MCV (*P* = 0.022). Details are available in Supplementary Table 6.

Finally, we compared the patients who died during the hospital stay with those who survived hospitalization and found that AP parameters regarding pupillary constriction (i.e. CH, CV, MCV) and dilation (i.e. DV), together with mean and homolateral MIN, and homolateral BPD were significantly lower in patients with poor prognosis (please refer to Supplementary Table 7). Details on the clinical, demographic and radiological comparisons between these groups are available in Supplementary Table 8.

### Adjusted analyses

We then performed adjusted analyses to evaluate predictors of 3-month stroke outcome, defined as mRS > 2, mRS > 3 and a shift in the direction of a worse outcome on the 3-month mRS. Based on the results of the univariate comparisons, we considered in the logistic regression model the following potential confounders: age, admission NIHSS, pre-morbid mRS, revascularization treatments, stroke volume, LVO, both DWM and PVWM Fazekas scores, haemorrhagic conversion, diabetes, concomitant use of β-blockers, together with each single AP parameter which was significantly different between groups in the univariate comparison. The VIF of the models ranged from 1.093 to 2.536, suggesting low collinearity.

**Figure 2:**
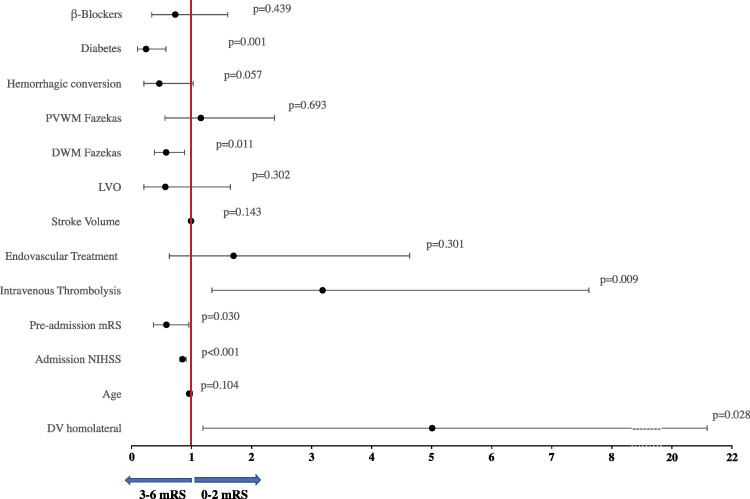
**The forest plot of the multivariable logistic regression to find independent predictors of 3-month mRS < 3 (*N* = 190).** mRS, modified Rankin Scale; PVWM, periventricular matter; DWM, deep white matter; LVO, large vessel occlusion; NIHSS, National Institute of Stroke Scale; DV, dilation velocity.

In both the dichotomizations of the mRS, pupillary DV in the eye homolateral to the ischaemic lesion was an independent predictor of stroke outcome (please refer to Table 3, Supplementary Table 9, Fig. 2). Furthermore, there was a significant shift in the distribution of 3-month mRS towards a worse outcome in the adjusted multivariable ordinal regression with the reduction of homolateral DV (common OR 0.308, 95% CI 0.103–0.925; *P* = 0.036).

**Table 3:** Results of multivariable logistic regression models predicting 3-month stroke outcome intended as mRS 0–2 versus mRS > 2, considering median ipsilateral DV

	mRS 0–2 versus mRS > 2	
	OR (95% confidence interval)	*P*-value
DV homolateral	5.013 (1.186–21.182)	**0**.**028**
Age	0.970 (0.934–1.006)	0.104
Admission NIHSS	0.852 (0.799–0.908)	**<0**.**001**
Pre-admission mRS	0.590 (0.366–0.950)	**0**.**030**
Intravenous Thrombolysis	3.194 (1.339–7.620)	**0**.**009**
Endovascular Treatment	1.699 (0.623–4.634)	0.301
Stroke volume	0.989 (0.983–0.995)	0.148
LVO	0.573 (0.199–1.650)	0.302
DWM Fazekas	0.583 (0.384–0.885)	**0**.**011**
PVWM Fazekas	1.156 (0.562–2.379)	0.693
Haemorrhagic conversion	0.461 (0.208–1.022)	0.057
Diabetes	0.240 (0.100–0.576)	**0**.**001**
β-Blockers	0.734 (0.336–1.606)	0.439

mRS, modified Rankin Scale; OR, odds ratio; DV, dilation velocity; LVO, large vessel occlusion; DWM, deep white matter; PVWM, periventricular matter. Significant findings are reported in bold.

On the other hand, none of the other AP parameters considered reached the statistical significance as independent predictors of study outcomes. Details are available in Supplementary Tables 10 and 11.

### Predictive performance

**Figure 3:**
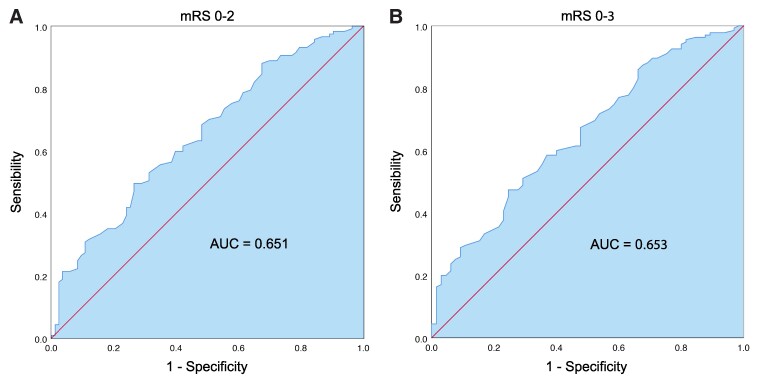
**The receiver operating characteristic (ROC) curves for the prediction of stroke outcomes based on homolateral DV values.** Panel (**A**): mRS 0–2 versus mRS > 2 dichotomization (*N* = 190). Panel (**B**): mRS 0–3 versus mRS > 3 dichotomization (*N* = 190). ROC, receiving operator characteristic; DV, dilatation velocity; mRS, modified Rankin Scale; AUC, area under the curve.

We then computed ROC curves to determine the ability of AP to predict stroke outcomes by considering the DV in the eye homolateral to the stroke lesion. Homolateral DV was found to be a good predictor of stroke outcome regarding both 3-month mRS 0–2 and mRS 0–3 (area under the curve, AUC: 0.651, CI (0.574–0.727), *P* < 0.001 and AUC: 0.653, CI (0.573–0.732), *P* < 0.001, respectively). Based on the Youden index, we found that the optimal cut-off for the mRS 0–2 versus mRS > 2 model was a DV of 0.865 mm/s, which has both a sensitivity and a specificity of 60% for outcome prediction. Similarly, for the mRS 0–3 versus mRS > 3 model, a cut-off of 0.845 mm/s could predict stroke outcome with 60% sensitivity and specificity (please refer to Fig. 3).

## Discussion

In this study, we found that several AP parameters recorded during the first 72 h after an ischaemic stroke may be useful in the prediction of 3-month stroke outcome. In particular, a reduction of DV in the eye homolateral to the ischaemic lesion has proven to be an independent predictor of poor 3-month outcome understood as both mRS > 2 and mRS > 3.

To date, no direct evidence is available regarding the prognostic role of AP in patients with acute ischaemic stroke. Previous studies were conducted on heterogeneous populations of critically ill patients with acute brain injury and found that NPi was the most sensitive AP parameter for predicting patient outcome.^18,20,30^ The only study focused on ischaemic stroke patients, found that mean CV, DV and CH were significantly lower in patients who developed radiological signs of brain oedema after mechanical thrombectomy for LVO compared with those without brain oedema.^24^

The results of our study suggest that NPi is not an independent predictor of in-hospital and 3-month outcome in patients with ischaemic stroke. It should be considered that previous studies included critically ill patients admitted to intensive care units, whose pathological NPi could be influenced by concomitant pharmacological treatments^31^ and by intracranial hypertension.^32,33^ In our population of patients with ischaemic stroke, we excluded patients with radiological evidence of intracranial hypertension and none received vasopressors or sedative agents during the stroke unit stay, suggesting that our population was mainly composed of non-critically ill patients. Furthermore, we did not include patients with posterior circulation strokes, thus we excluded subjects with pupillary dysfunction due to a direct lesion of the PLR pathways. Consequently, we can hypothesize that the observed alterations of the pupillary reactivity in our patients may be mediated by mechanisms other than mechanical compression or an ischaemic brainstem lesion. In support of this hypothesis, a recent study reported that NPi failed to be an independent predictor of delayed cerebral ischaemia in patients with subarachnoid haemorrhage.^9^ These data could be explained considering that NPi is influenced almost exclusively by the brainstem function, making it a useful measure for early detection of intracranial hypertension,^14,34^ while it is only marginally influenced by cortical activity, contrary to other AP variables.^13^

The PLR is an ancient, evolutionary-conserved reflex mediated by both branches of the autonomic nervous system which exert opposite functions: pupillary constriction is mediated, through a by-synaptic reflex, by the parasympathetic nervous system, while the sympathetic branch induces pupil dilatation by means of a three-neuron arch.^35^ Current knowledge suggests that, while the parasympathetic circuit of pupillary function is principally under a subcortical control, the sympathetic arch is largely influenced by cortical inputs.^10,36^ Pupillary dilatation occurs during several cognitive processes and is altered by many emotional stimuli.^10^ Lesions in strategic brain regions, such as the insular cortex, frontal eyes fields and amygdala, have been associated with an impairment of pupillary dilatation in humans and in non-human primates, and the electrical stimulation of the frontal cortex induces pupil dilatation in animal models.^10^ Furthermore, a recent study found that the only PLR parameter altered by strategic cortical infarctions (i.e. ischaemic lesions of the frontal eye fields and/or insula) was the loss of the correlation of pupil size and DV in right-hemispheric lesions.^13^

The results of our study reporting an impairment of the DV in the eye homolateral to the ischaemic lesion support previous evidence on the cortical modulation of this dynamic pupillary parameter and suggest that an impairment of the ipsilateral sympathetic cortical control of pupillary function leads to a worse 3-month outcome of patients with ischaemic stroke.

Autonomic dysfunction is a well-known consequence of acute stroke, impacting up to 76% of stroke patients,^37^ and which has been largely associated with a worse prognosis, both in terms of mortality and long-term functional disability.^37,38^ However, to date, the available evidence focused on the cardiovascular features of dysautonomia, highlighting how lesions of the right hemisphere, predominantly affecting the insular cortex, are associated, to stroke-related cardiovascular dysautonomia, inducing a sympathetic withdrawal and a parasympathetic hypertonus.^38^ However, the exact mechanisms by which autonomic impairment can lead to worse functional outcomes are not well understood. One hypothesis is that dysautonomia may increase the blood pressure variability in stroke patients, leading to sustained cerebral hypoperfusion and, consequently, neurological deterioration and low compliance with rehabilitation.^37,39^ Moreover, abnormal autonomic control has been established to independently predict morbidity and death due to cardiovascular reasons.^40^ The results of our study support this evidence, as we confirmed that an early sympathetic impairment leads to a poor in-hospital and 3-month prognosis.

This is the first study to suggest the role of AP as a rapid, non-invasive, and useful tool to predict the 3-month outcome of patients with acute ischaemic stroke. In contrast with previous literature, we included non-critically ill patients, finding that AP could be useful in detecting subtle changes of pupillary reactivity linked to stroke prognosis, regardless of intracranial hypertension or extensive brain injury. In a rapidly changing system of care where artificial intelligence would soon be able to give diagnostic and prognostic prediction based on the analysis of a big amount of data, DV is a potential candidate as a further independent and objective parameter to be included in computational analyses.

The limitations of the study are as follows: (i) the single-centre design; (ii) the relatively small sample sizes, in particular for intra-hospital mortality; (iii) AP analysis, although in triplicate, under conditions replicable for ambient brightness, hospitalization site, time distance from the index event and the circadian time, was carried out in a single survey and (iv) multiple testing.

Due to these limitations, further multi-centric studies, conducted on larger samples, would be useful to evaluate the AP over time and possibly correlate it to the clinical evolution of patients in order to confirm the results of our study.

## Conclusion

Ischaemic strokes cause an early alteration in pupillary dynamics suggestive of sympathetic withdrawal which could be easily detected by AP performed in the first days after stroke onset. In particular, the reduction in DV of the eye ipsilateral to the ischaemic lesion could be an independent predictor of unfavourable short- and long-term outcomes and could therefore already be used in the emergency department to identify ischaemic stroke patients who require more intensive monitoring and a personalized pathway.

## Supplementary Material

fcaf079_Supplementary_Data

## Data Availability

The raw data available upon reasonable request from the corresponding author. No codes were generated or used in this work.
