# Supplementary material for: Automated pupillometry is a predictor of outcome of stroke patients: an observational, prospective, cohort study
Source: Brain Commun. 2025 Feb 18;7(1):fcaf079. doi: 10.1093/braincomms/fcaf079 (PMC11878543; doi:10.1093/braincomms/fcaf079)
Supplement: fcaf079_Supplementary_Data [file fcaf079_supplementary_data.docx]

**Supplementary Materials**

**Supplementary Table 1.** A list of the abbreviations included in the manuscript.

| AP | Automated Pupillometry |
| --- | --- |
| mRS | modified Rankin Scale |
| NPi | Neurological Pupil Index |
| DV | Dilation Velocity |
| PLR | Pupillary Light Reflex |
| STROBE | Strengthening the Reporting of Observational Studies in Epidemiology |
| NIHSS | National Institute of Health Stroke Scale |
| TOAST | Trial of Org 10172 in Acute Stroke Treatment |
| CT | Computed Tomography |
| MRI | Magnetic Resonance Imaging |
| PVWM | PeriVentricular White Matter |
| DWM | Deep White Matter |
| LVO | Large Vessel Occlusion |
| ROC | Receiver Operating Characteristic |
| ICC | Intraclass Correlation Coefficient |
| CI | Confidence Interval |
| DV | Dilation Velocity |
| CV | Average Constriction Velocity |
| MCV | Maximum Constriction Velocity |
| MIN | Minimum Pupil diam |
| BPD | Baseline Pupil Diameter |
| CH | Percentage of constriction |
| LAT | Latency of constriction |
| AUC | Area Under the Curve |

|  | **Abbreviations** | **UoM** | **Definition** | **ANS branch** |
| --- | --- | --- | --- | --- |
| **Baseline Pupil Diameter** | BPD | mm | Maximum pupillary diameter at baseline, before the light stimulus | Both |
| **Minimum Pupil Diameter** | MIN | mm | Pupillary diameter at peak constriction | Parasympathetic |
| **Reflex Latency** | LAT | s | Time delay between the light stimulus and the onset of pupillary constriction | Parasympathetic |
| **Average Constriction Velocity** | CV | mm/s | Average velocity of the pupil constriction | Parasympathetic |
| **Maximum Constriction Velocity** | MCV | mm/s | Maximum velocity of the pupil constriction | Parasympathetic |
| **%Change – Percentage of Constriction** | CH | % | Obtained by applying the sequent formula: (Size -MIN)/Size*100 | Parasympathetic |
| **Dilation Velocity** | DV | mm/s | Average velocity of the pupil dilation | Both, mostly sympathetic |
| **Neurological Pupil Index** | NPi | N/A | A composite measure obtained by the combination of all the aforementioned parameters | Both |

**Supplementary Table 2.** A descriptive table reassuming the parameters collected by NPi-200^®^ (NeurOptics, Irvine, CA, USA). Abbreviations: UoM, Unit of Measurement; ANS, Autonomic Nervous System

**Supplementary Table 3**. Univariate comparison of demographics, clinical and radiological parameters between patients with 3-month mRS 0-3 vs those with mRS>3 at three months.

|  | mRS 0-3  n = 143 | mRS 4-6  n =66 | p |
| --- | --- | --- | --- |
| Age (years) | 71.00 (60.00 - 80.00) | 80.00 (72.50 - 86.00) | **<0.001** |
| Male sex | 90 (62.94%) | 33 (50.00%) | 0.077 |
| Pre-admission mRS | 0.00 (0.00 - 1.00) | 0.50 (0.00 - 1.00) | **0.012** |
| Admission NIHSS | 4.00 (2.00 - 9.00) | 11.50 (6.00 - 20.25) | **<0.001** |
| Intravenous Thrombolysis | 50 (34.97%) | 17 (25.76%) | 0.185 |
| Endovascular Treatment | 46 (32.17%) | 33 (50.00%) | **0.013** |
|  |  |  |  |
| Radiological features |  |  |  |
| LVO | 72 (50.35%) | 51 (77.27%) | **<0.001** |
| Stroke Volume (cm^3^) | 2.53 (0.61 – 10.69) | 27.34 (6.81 – 105.36) | **<0.001** |
| DWM Fazekas | 1.00 (0.00 - 2.00) | 1.00 (1.00 - 2.00) | **0.006** |
| PVM Fazekas | 1.00 (0.00 - 2.00) | 2.00 (1.00 - 2.00) | **0.001** |
| Hemorrhagic conversion | 34 (23.78%) | 31 (46.97%) | **0.001** |
|  |  |  |  |
| TOAST classification |  |  |  |
| Large artery atherosclerosis | 25 (17.48%) | 16 (24.24%) | 0.151 |
| Cardioembolism | 38 (26.57%) | 25 (37.88%) |  |
| Lacunar | 31 (21.68%) | 7 (10.61%) |  |
| Other determined etiologies | 12 (8.39%) | 4 (6.06%) |  |
| Cryptogenic | 36 (25.17%) | 12 (18.18%) |  |
|  |  |  |  |
| Comorbidities |  |  |  |
| Diabetes | 24 (16.78%) | 26 (39.39%) | **<0.001** |
| Hypertension | 113 (79.02%) | 56 (84.85%) | 0.32 |
| Dyslipidemia | 64 (44.76%) | 30 (45.45%) | 0.925 |
| Obesity | 24 (16.78%) | 11 (16.67%) | 0.983 |
| Smoking habit | 40 (27.97%) | 11 (16.67%) | 0.077 |
| Former smokers | 9 (6.29%) | 3 (4.55%) | 0.615 |
| Current smokers | 31 (21.68%) | 8 (12.12%) | 0.099 |
| Atrial Fibrillation | 29 (20.28%) | 24 (36.36%) | 0.013 |
| Cancer | 21 (14.69%) | 12 (18.18%) | 0.519 |
| Liver disease | 3 (2.10%) | 2 (3.03%) | 0.682 |
| Kidney Failure | 11 (7.69%) | 8 (12.12%) | 0.301 |
|  |  |  |  |
| Concomitant drugs |  |  |  |
| β-Blockers | 57 (39.86%) | 39 (59.09%) | **0.01** |
| α-Blockers | 23 (16.08%) | 8 (12.12%) | 0.454 |
| ACE-inhibitors | 69 (48.25%) | 37 (56.06%) | 0.294 |
| Sartans | 36 (25.17%) | 12 (18.18%) | 0.264 |
| Calcium Channel Blockers | 48 (33.57%) | 50 (75.76%) | 0.225 |
| SSRI | 7 (4.90%) | 2 (3.03%) | 0.537 |
|  |  |  |  |
| Outcome parameters |  |  |  |
| Length of hospital stay (days) | 7.00 (5.00 - 9.00) | 7.00 (5.00 - 15.00) | 0.261 |
| Discharge NIHSS | 1.00 (0.00 - 3.00) | 9.00 (4.00 - 17.25) | **<0.001** |
| Discharge mRS | 1.00 (1.00 - 2.00) | 4.00 (4.00 - 5.00) | **<0.001** |

*Abbreviations: mRS, modified Rankin Scale; NIHSS, National Institute of Health Stroke Scale; LVO, Large Vessel Occlusion; DWM, Deep White Matter; PVM, Periventricular Matter; ACE, Angiotensin Converting Enzyme; SSRIs, Selective serotonin reuptake inhibitors*

**Supplementary Table 4.** Table depicting the univariate comparisons of AP parameters between patients with a three-month mRS of 0-3 vs those with a mRS>3.

| Automated Pupillometry | mRS 0-3  n = 143 | mRS 4-6  n =66 | p |
| --- | --- | --- | --- |
| NPi mean | 4.55 (4.35 - 4.72) | 4.55 (4.30 - 4.70) | 0.741 |
| NPi homolateral | 4.60 (4.40 - 4.70) | 4.60 (4.39 - 4.72) | 0.796 |
| NPi contralateral | 4.53 (4.30 - 4.70) | 4.53 (4.30 - 4.70) | 0.738 |
| NPi absolute difference | 0.10 (0.07 – 0.30) | 0.17 (0.07 – 0.30) | 0.311 |
|  |  |  |  |
| BPD mean (mm) | 3.34 (2.80 – 3.81) | 3.19 (2.73 – 3.67) | 0.184 |
| BPD homolateral (mm) | 3.32 (2.81 – 3.83) | 3.29 (2.64 - 3.59) | 0.161 |
| BPD contralateral (mm) | 3.33 (2.86 – 3.95) | 3.12 (2.61 – 3.73) | 0.100 |
| BPD absolute difference (mm) | 0.29 (0.12 – 0.50) | 0.36 (0.15 – 0.59) | 0.101 |
|  |  |  |  |
| MIN mean (mm) | 2.35 (2.04 – 2.64) | 2.26 (1.94 - 2.51) | 0.132 |
| MIN homolateral (mm) | 2.32 (2.00 – 2.63) | 2.29 (1.89 - 2.50) | 0.166 |
| MIN contralateral (mm) | 2.33 (2.01 – 2.65) | 2.27 (1.97 - 2.56) | 0.186 |
| MIN absolute difference (mm) | 0.15 (0.07 – 0.29) | 0.20 (0.07 – 0.32) | 0.394 |
|  |  |  |  |
| CH mean (%) | 30.00 (24.50 – 35.00) | 30.00 (21.00 - 33.13) | 0.135 |
| CH homolateral (%) | 30.00 (25.00 – 35.00) | 30.00 (25.00 - 34.00) | 0.557 |
| CH contralateral (%) | 31.00 (24.00 – 36.00) | 28.00 (20.00 – 35.00) | **0.011** |
| CH absolute differences (%) | 3.00 (2.00 – 6.00) | 4.50 (2.00 – 8.00) | **0.027** |
|  |  |  |  |
| CV mean (mm/s) | 1.98 (1.54 – 2.42) | 1.89 (1.19 – 2.27) | **0.034** |
| CV homolateral (mm/s) | 1.91 (1.52 - 2.36) | 1.81 (1.29 – 2.36) | 0.193 |
| CV contralateral (mm/s) | 2.03 (1.57 – 2.49) | 1.78 (1.06 – 2.28) | **0.008** |
| CV absolute difference (mm/s) | 0.30 (0.12 – 0.56) | 0.34 (0.19 – 0.68) | 0.234 |
|  |  |  |  |
| MCV mean (mm/s) | 2.97 (2.29 - 3.65) | 2.85 (1.75 – 3.44) | 0.091 |
| MCV homolateral (mm/s) | 2.86 (2.23 - 3.55) | 2.85 (2.00 – 3.50) | 0.265 |
| MCV contralateral (mm/s) | 2.96 (2.28 – 3.82) | 2.71 (1.70 – 3.51) | **0.011** |
| MCV absolute difference (mm/s) | 0.38 (0.14 – 0.75) | 0.47 (0.17 – 0.82) | 0.351 |
|  |  |  |  |
| LAT mean (s) | 0.240 (0.215 - 0.270) | 0.250 (0.219 - 0.270) | 0.977 |
| LAT homolateral (s) | 0.230 (0.200 - 0.270) | 0.230 (0.215 - 0.270) | 0.862 |
| LAT contralateral (s) | 0.230 (0.230 - 0.270) | 0.240 (0.230 - 0.270) | 0.393 |
| LAT absolute difference (s) | 0.30 (0.00 – 0.30) | 0.30 (0.00 – 0.43) | 0.325 |
|  |  |  |  |
| DV mean (mm/s) | 0.92 (0.77 – 1.13) | 0.81 (0.61 – 0.95) | **<0.001** |
| DV homolateral (mm/s) | 0.95 (0.72 – 1.13) | 0.78 (0.60 – 0.99) | **<0.001** |
| DV contralateral (mm/s) | 0.94 (0.73 – 1.11) | 0.78 (0.58 – 0.97) | **<0.001** |
| DV absolute difference (mm/s) | 0.14 (0.07 – 0.23) | 0.16 (0.07 – 0.25) | 0.669 |

*Abbreviations: AP, Automated Pupillometry; mRS, modified Rankin Scale; NPi, Neurological Pupil Index; BPD, Baseline Pupil Diameter; MIN, Minimum pupil diameter; CH, Percentage of Constriction; CV, average Constriction Velocity; MCV, Maximum Constriction Velocity; LAT, reflex LATency; DV, Dilation Velocity.*

**Supplementary Table 5.** The univariate comparison of clinical parameters between deceased patients and those who survived at three months

|  | 3-month mRS 0-5  (n = 181) | 3-month mRS 6  (n = 28) | p |
| --- | --- | --- | --- |
| Age (years) | 73.00 (61.50 - 81.50) | 82.00 (73.50 – 88.50) | **<0.001** |
| Male sex | 110 (60.77%) | 13 (46.43%) | 0.151 |
| Pre-admission mRS | 0.00 (0.00 - 1.00) | 0.00 (1.00 - 2.00) | **0.001** |
| Admission NIHSS | 5.00 (2.00 - 10.75) | 18.0 (8.25 - 22.75) | **<0.001** |
| Intravenous Thrombolysis | 58 (32.04%) | 9 (32.14%) | 0.992 |
| Endovascular Treatment | 64 (36.36%) | 15 (53.57%) | 0.064 |
|  |  |  |  |
| Radiological features |  |  |  |
| LVO | 101 (55.80%) | 22 (78.57%) | **0.011** |
| Stroke Volume (cm^3^) | 3.43 (0.73 – 19.74) | 69.97 (21.19 – 201.80) | **<0.001** |
| DWM Fazekas | 1.00 (0.00 - 2.00) | 1.00 (1.00 - 2.00) | 0.240 |
| PVM Fazekas | 1.00 (1.00 - 2.00) | 2.00 (1.00 - 2.00) | **0.019** |
| Hemorrhagic conversion | 49 (27.07%) | 16 (57.14%) | **0.001** |
|  |  |  |  |
| TOAST classification |  |  |  |
| Large artery atherosclerosis | 35 (19.34%) | 6 (21.43%) | 0.146 |
| Cardioembolism | 50 (27.62%) | 13 (46.43%) |  |
| Lacunar | 36 (19.89%) | 2 (7.14%) |  |
| Other determined etiologies | 13 (7.18%) | 3 (10.7%) |  |
| Cryptogenic | 47 (25.97%) | 4 (14.29%) |  |
|  |  |  |  |
| Comorbidities |  |  |  |
| Diabetes | 39 (21.55%) | 11 (39.29%) | **0.041** |
| Hypertension | 143 (79.01%) | 26 (92.86%) | 0.083 |
| Dyslipidemia | 85 (46.96%) | 9 (32.14%) | 0.142 |
| Obesity | 29 (16.02%) | 6 (21.43%) | 0.476 |
| Smoking habit | 46 (25.70%) | 5 (17.86%) | 0.420 |
| Former Smokers | 10 (5.52%) | 2 (7.14%) | 0.707 |
| Current Smokers | 36 (19.89%) | 3 (10.71%) | 0.266 |
| Atrial Fibrillation | 41 (22.65%) | 12 (42.86%) | **0.022** |
| Cancer | 28 (15.47%) | 5 (17.86%) | 0.747 |
| Liver disease | 4 (2.21%) | 1 (3.57%) | 0.661 |
| Kidney Failure | 12 (6.63%) | 7 (25.00%) | **0.002** |
|  |  |  |  |
| Concomitant drugs |  |  |  |
| β-Blockers | 77 (42.54%) | 19 (67.86%) | **0.012** |
| α-Blockers | 30 (16.57%) | 1 (3.57%) | 0.072 |
| ACE-inhibitors | 93 (51.38%) | 13 (46.43%) | 0.626 |
| Sartans | 43 (23.76%) | 5 (17.86%) | 0.490 |
| Calcium Channel Blockers | 88 (48.62%) | 10 (35.71%) | 0.925 |
| SSRI | 8 (4.42%) | 1 (3.57%) | 0.837 |
| Tryciclic antidepressant | 0 (0%) | 0 (0%) | NA |
|  |  |  |  |
| Outcome parameters |  |  |  |
| Length of hospital stay (days) | 7.00 (6.00 - 10.00) | 7.40 (4.00 - 15.75) | 0.261 |
| Discharge NIHSS | 2.00 (0.00 - 4.00) | 18.00 (7.00 - 42.00) | **<0.001** |
| Discharge mRS | 2.00 (1.00 - 3.00) | 5.00 (4.75 - 6.00) | **<0.001** |

*Abbreviations: mRS, modified Rankin Scale; NIHSS, National Institute of Health Stroke Scale; LVO, Large Vessel Occlusion; DWM, Deep White Matter; PVM, Periventricular Matter; ACE, Angiotensin Converting Enzyme; SSRIs, Selective serotonin reuptake inhibitors*

**Supplementary Table 6.** Univariate comparison of AP parameters between patients alive and those deceased at the 3-month follow-up.

| Automated Pupillometry | 3-month mRS 0-5  n = 181 | 3-month mRS 6  n =28 | p |
| --- | --- | --- | --- |
| NPi mean | 4.55 (4.35 - 4.70) | 4.62 (4.30 - 4.73) | 0.497 |
| NPi homolateral | 4.40 (4.60 - 4.70) | 4.62 (4.32 - 4.80) | 0.826 |
| NPi contralateral | 4.57 (4.30 - 4.70) | 4.50 (4.10 - 4.69) | 0.153 |
| NPi absolute difference | 0.10 (0.07 – 0.29) | 0.12 (0.04 – 0.38) | 0.061 |
|  |  |  |  |
| BPD mean (mm) | 3.32 (2.80 – 3.79) | 3.13 (2.49 – 3.69) | 0.179 |
| BPD homolateral (mm) | 3.34 (2.81 – 3.80) | 3.08 (2.46 - 3.45) | **0.047** |
| BPD contralateral (mm) | 3.29 (2.85 – 3.88) | 3.24 (2.49 – 3.81) | 0.363 |
| BPD absolute difference (mm) | 0.29 (0.13 – 0.53) | 0.39 (0.20 – 0.61) | 0.097 |
|  |  |  |  |
| MIN mean (mm) | 2.34 (2.01 – 2.62) | 2.18 (1.88 - 2.53) | 0.148 |
| MIN homolateral (mm) | 2.32 (2.00 – 2.63) | 2.15 (1.85 - 2.41) | **0.045** |
| MIN contralateral (mm) | 2.32 (2.00 – 2.64) | 2.21 (1.87 - 2.65) | 0.571 |
| MIN absolute difference (mm) | 0.16 (0.07 – 0.29) | 0.21 (0.63 – 0.37) | 0.461 |
|  |  |  |  |
| CH mean (%) | 30.50 (24.50 – 35.00) | 26.75 (21.00 – 32.75) | **0.013** |
| CH homolateral (%) | 30.00 (25.00 – 35.00) | 29.00 (22.75 – 32.75) | 0.067 |
| CH contralateral (%) | 30.00 (24.00 - 36.00) | 25.50 (18.25 – 33.75) | **0.019** |
| CH absolute difference (%) | 4.00 (2.00 – 6.50) | 3.50 (2.00 – 8.25) | 0.970 |
|  |  |  |  |
| CV mean (mm/s) | 1.98 (1.53 – 2.42) | 1.64 (1.09 – 2.19) | **0.012** |
| CV homolateral (mm/s) | 1.92 (1.51 – 2.40) | 1.69 (1.12 – 2.05) | **0.035** |
| CV contralateral (mm/s) | 2.02 (1.52 – 2.45) | 1.67 (0.90 – 2.32) | **0.011** |
| CV absolute difference (mm/s) | 0.30 (0.14 – 0.58) | 0.34 (0.22 – 0.72) | 0.394 |
|  |  |  |  |
| MCV mean (mm/s) | 2.98 (2.28 – 3.61) | 2.50 (1.61 – 3.30) | **0.019** |
| MCV homolateral (mm/s) | 2.86 (2.22 – 3.57) | 2.57 (1.73 – 3.39) | **0.022** |
| MCV contralateral (mm/s) | 2.96 (2.21 – 3.75) | 2.39 (1.30 – 3.50) | **0.012** |
| MCV absolute difference (mm/s) | 0.42 (0.15 – 0.77) | 0.37 (0.10 – 0.77) | 0.770 |
|  |  |  |  |
| LAT mean (s) | 0.240 (0.215 - 0.270) | 0.250 (0.215 - 0.270) | 0.870 |
| LAT homolateral (s) | 0.230 (0.200 - 0.270) | 0.240 (0.202 - 0.270) | 0.540 |
| LAT contralateral (s) | 0.230 (0.230 - 0.270) | 0.250 (0.230 - 0.270) | 0.175 |
| LAT absolute difference (s) | 0.030 (0.00 – 0.040) | 0.020 (0.00 – 0.040) | 0.810 |
|  |  |  |  |
| DV mean (mm/s) | 0.90 (0.73 – 1.10) | 0.82 (0.57 – 0.94) | **0.003** |
| DV homolateral (mm/s) | 0.89 (0.70 – 1.10) | 0.80 (0.59 – 0.92) | **0.005** |
| DV contralateral (mm/s) | 0.91 (0.69 – 1.10) | 0.78 (0.56 – 0.99) | **0.012** |
| DV absolute difference (mm/s) | 0.15 (0.07 – 0.24) | 0.12 (0.06 – 0.23) | 0.527 |

*Abbreviations: mRS, modified Rankin Scale; NPi, Neurological Pupil Index; BPD, Baseline Pupil Diameter; MIN, Minimum pupil diameter; CH, Percentage of Constriction; CV, average Constriction Velocity; MCV, Maximum Constriction Velocity; LAT, reflex LATency; DV, Dilation Velocity.*

**Supplementary Table 7.** A univariate comparison between patients who died and those who survived during the hospital admission.

| Automated Pupillometry | Discharge mRS 0-5 (n = 198) | Discharge mRS 6 (n =11) | p |
| --- | --- | --- | --- |
| NPi mean | 4.55 (4.35 - 4.70) | 4.30 (3.95 – 4.75) | 0.256 |
| NPi homolateral | 4.60 (4.40 - 4.70) | 4.50 (4.00 – 4.80) | 0.878 |
| NPi contralateral | 4.57 (4.30 - 4.70) | 4.13 (3.90 – 4.70) | *0.072* |
| NPi absolute difference | 0.10 (0.07 – 0.28) | 0.20 (0.10 – 0.40) | 0.147 |
|  |  |  |  |
| BPD mean (mm) | 3.32 (2.82 – 3.78) | 2.56 (2.27 – 3.70) | 0.060 |
| BPD homolateral (mm) | 3.29 (2.81 - 3.76) | 2.56 (1.98 – 3.68) | **0.046** |
| BPD contralateral (mm) | 3.30 (2.85 – 3.88) | 2.99 (2.14 – 3.72) | 0.0150 |
| BPD absolute difference (mm) | 0.30 (0.14 – 0.53) | 0.47 (0.12 – 0.61) | 0.398 |
|  |  |  |  |
| MIN mean (mm) | 2.34 (2.01 – 2.61) | 1.88 (1.70 – 2.53) | **0.005** |
| MIN homolateral (mm) | 2.32 (2.00 – 2.61) | 1.75 (1.48 – 2.42) | **0.002** |
| MIN contralateral (mm) | 2.32 (2.01 - 2.64) | 2.12 (1.61 – 2.65) | 0.168 |
| MIN absolute difference (mm) | 0.16 (0.07 – 0.29) | 0.23 (0.06 – 0.35) | 0.478 |
|  |  |  |  |
| CH mean (%) | 30.50 (24.00 – 35.00) | 23.50 (11.00 – 30.00) | **<0.001** |
| CH homolateral (%) | 30.00 (25.00 – 35.00) | 26.00 (12.00 – 30.00) | **0.003** |
| CH contralateral (%) | 30.00 (24.00 - 36.00) | 20.00 (9.00 – 29.00) | **0.004** |
| CH absolute difference (%) | 4.00 (2.00 – 6.00) | 4.00 (2.00 – 10.00) | 0.482 |
|  |  |  |  |
| CV mean (mm/s) | 1.98 (1.50 – 2.40) | 1.20 (0.70 – 2.15) | **0.003** |
| CV homolateral (mm/s) | 1.91 (1.51 – 2.39) | 1.53 (0.94 – 2.00) | **0.015** |
| CV contralateral (mm/s) | 2.03 (1.48 – 2.45) | 0.86 (0.51 – 1.87) | **0.002** |
| CV absolute difference (mm/) | 0.30 (0.14 – 0.58) | 0.43 (0.23 – 0.73) | 0.554 |
|  |  |  |  |
| MCV mean (mm/s) | 2.98 (2.26 - 3.60) | 1.67 (1.00 – 2.87) | **0.010** |
| MCV homolateral (mm/s) | 2.86 (2.19 – 3.55) | 2.12 (1.29 – 3.04) | **0.006** |
| MCV contralateral (mm/s) | 2.96 (2.20 – 3.74) | 1.22 (0.78 – 2.65) | **0.002** |
| MCV absolute difference (mm/s) | 0.41 (0.15 – 0.76) | 0.59 (0.03 – 0.90) | 0.840 |
|  |  |  |  |
| LAT mean (s) | 0.250 (0.215 - 0.270) | 0.230 (0.215 – 0.250) | 0.256 |
| LAT homolateral (s) | 0.230 (0.200 - 0.270) | 0.230 (0.210 – 0.260) | 0.158 |
| LAT contralateral (s) | 0.230 (0.230 - 0.270) | 0.240 (0.230 – 0.270) | 0.830 |
| LAT absolute difference (s) | 0.030 (0.000 – 0.040) | 0.020 (0.000 – 0.040) | 0.432 |
|  |  |  |  |
| DV mean (mm/s) | 0.90 (0.73 – 1.08) | 0.56 (0.28 – 0.77) | **<0.001** |
| DV homolateral (mm/s) | 0.88 (0.70 – 1.09) | 0.59 (0.29 – 0.84) | **<0.001** |
| DV contralateral (mm/s) | 0.91 (0.71 – 1.09) | 0.53 (0.27 – 0.67) | **<0.001** |
| DV absolute difference (mm/s) | 0.15 (0.07 – 0.24) | 0.08 (0.02 – 0.22) | 0.117 |

*Abbreviations: mRS, modified Rankin Scale; NPi, Neurological Pupil Index; BPD, Baseline Pupil Diameter; MIN, Minimum pupil diameter; CH, Percentage of Constriction; CV, average Constriction Velocity; MCV, Maximum Constriction Velocity; LAT, reflex LATency; DV, Dilation Velocity.*

**Supplementary Table 8**. Univariate comparison of demographics, clinical and radiological parameters between patients who died and those who survived during the hospital stay.

|  | mRS 0-5 at discharge (n = 198) | In-hospital death (n = 11) | p |
| --- | --- | --- | --- |
| Age (years) | 75.00 (63.00 - 82.00) | 82.00 (69.00 – 87.00) | **0.022** |
| Male sex | 117 (50.09%) | 6 (54.55%) | 0.763 |
| Pre-admission mRS | 0.00 (0.00 - 1.00) | 0.00 (0.00 - 3.00) | **0.021** |
| Admission NIHSS | 5.00 (2.00 - 12.00) | 21.00 (11.00 – 23.00) | **<0.001** |
| Intravenous Thrombolysis | 64 (32.32%) | 3 (27.27%) | >0.99 |
| Endovascular Treatment | 74 (37.37%) | 5 (45.50%) | 0.751 |
|  |  |  |  |
| Radiological features |  |  |  |
| LVO | 114 (57.58%) | 9 (81.82%) | 0.206 |
| Stroke Volume (cm^3^) | 4.51 (0.83 – 23.05) | 205.743 (118.9 – 307.91) | **<0.001** |
| DWM Fazekas | 1.00 (0.00 - 2.00) | 1.50 (0.75 - 2.00) | 0.676 |
| PVM Fazekas | 1.00 (1.00 - 2.00) | 2.00 (1.00 - 2.25) | 0.142 |
| Hemorrhagic conversion | 57 (28.79%) | 8 (72.73%) | **0.004** |
|  |  |  |  |
| TOAST classification |  |  |  |
| Large artery atherosclerosis | 37 (18.69%) | 4 (36.36%) | 0.303 |
| Cardioembolism | 59 (29.80%) | 4 (36.36%) |  |
| Lacunar | 38 (19.19%) | 0 (0%) |  |
| Other determined etiologies | 16 (8.08%) | 0 (0%) |  |
| Cryptogenic | 48 (24.24%) | 3 (27.27%) |  |
|  |  |  |  |
| Comorbidities |  |  |  |
| Diabetes | 46 (23.23%) | 4 (36.36%) | 0.299 |
| Hypertension | 158 (79.80%) | 11 (100%) | 0.129 |
| Dyslipidemia | 92 (46.46%) | 2 (18.18%) | 0.116 |
| Obesity | 32 (16.16%) | 3 (27.27%) | 0.399 |
| Smoking habit | 49 (24.75%) | 2 (18.18%) | >0.99 |
| Former smokers | 11 (5.56%) | 1 (9.09%) | 0.459 |
| Current smokers | 38 (19.19%) | 1 (9.09%) | 0.403 |
| Atrial Fibrillation | 49 (24.74%) | 4 (36.36%) | 0.476 |
| Cancer | 33 (16.67%) | 0 (0%) | 0.219 |
| Liver disease | 5 (2.53%) | 0 (0%) | <0.99 |
| Kidney Failure | 17 (8.59%) | 2 (18.18%) | 0.263 |
|  |  |  |  |
| Concomitant drugs |  |  |  |
| β-Blockers | 90 (45.45%) | 6 (54.55%) | 0.758 |
| α-Blockers | 31 (15.66%) | 0 (0%) | 0.375 |
| ACE-inhibitors | 103 (52.02%) | 3 (27.27%) | 0.131 |
| Sartans | 46 (23.23%) | 2 (18.18%) | >0.99 |
| Calcium Channel Blockers | 95 (47.98%) | 3 (27.27%) | 0.816 |
| SSRI | 9 (4.55%) | 0 (0%) | >0.99 |

|  |  |  |  |
| --- | --- | --- | --- |

*Abbreviations: mRS, modified Rankin Scale; NIHSS, National Institute of Health Stroke Scale; LVO, Large Vessel Occlusion; DWM, Deep White Matter; PVM, Periventricular Matter; ACE, Angiotensin Converting Enzyme; SSRIs, Selective serotonin reuptake inhibitors*

**Supplementary Table 9.** Results of multivariable logistic regression models predicting 3-month stroke outcome intended as mRS 0-3 vs mRS>3, considering median ipsilateral DV.

|  |  | mRS 0-3 vs mRS>3 | |
| --- | --- | --- | --- |
|  |  | **OR (95% confidence interval)** | **p-value** |
| DV homolateral |  | 6.033 (1.262 - 28.853) | **0.024** |
| Age |  | 0.966 (0.925 - 1.010) | 0.128 |
| Admission NIHSS |  | 0.864 (0.804 - 0.927) | **<0.001** |
| Pre-admission mRS |  | 1.138 (0.689 - 1.882) | 0.613 |
| Intravenous Thrombolysis |  | 4.561 (1.607 - 12.940) | **0.004** |
| Endovascular Treatment |  | 1.334 (0.455 - 3.907) | 0.599 |
| Stroke Volume |  | 0.982 (0.970 - 0.994) | **0.003** |
| LVO |  | 0.650 (0.203 - 2.077) | 0.467 |
| DWM Fazekas |  | 0.774 (0.414 - 1.448) | 0.423 |
| PVM Fazekas |  | 0.411 (0.242 - 0.700) | **0.001** |
| Hemorrhagic conversion |  | 0.528 (0.216 - 1.288) | 0.16 |
| Diabetes |  | 0.179 (0.071 - 0.450) | **0.001** |
| β-Blockers |  | 0.910 (0.390 - 2.127) | 0.828 |

*Abbreviations: mRS, modified Rankin Scale; OR, Odds Ratio; DV, Dilation Velocity; LVO, Large Vessel Occlusion; DWM, Deep White Matter; PVM, PeriVentricular Matter.*

**Supplementary Table 10.** The results of the multivariable logistic regression models for the prediction of 3-month stroke outcome, intended both as mRS 0-2 vs mRS>2 and as mRS 0-3 vs mRS>3. All the AP parameters were adjusted for admission NIHSS, premorbid mRS, revascularization treatments, stroke volume, LVO, both DWM, and PVM Fazekas scores, hemorrhagic conversion, diabetes, and the concomitant use of β-blockers.

|  | mRS 0-2 vs mRS>2 | |  | mRS 0-3 vs mRS>3 | |
| --- | --- | --- | --- | --- | --- |
|  | **OR (95% confidence interval)** | **p-value** |  | **OR (95% confidence interval)** | **p-value** |
| CV mean | 1.215 (0.658 - 2.244) | 0.533 |  | 1.090 (0.561 - 2.117) | 0.799 |
| CV homolateral | 1.191 (0.647 - 2.193) | 0.574 |  |  |  |
| CV contralateral | 1.211 (0.707 - 2.074) | 0.486 |  | 1.208 (0.686 - 2.127) | 0.514 |
| MCV mean | 1.133 (0.753 - 1.705) | 0.550 |  |  |  |
| MCV contralateral | 1.098 (0.767 - 1.572) | 0.608 |  | 1.075 (0.736 - 1.570) | 0.709 |
| CH contralateral | 0.997 (0.952 - 1.045) | 0.904 |  | 1.006 (0.959 - 1.055) | 0.810 |
| DV contralateral | 1.600 (0.375 - 6.826) | 0.525 |  | 1.995 (0.452 - 8.815) | 0.362 |
| DV mean | 2.180 (0.433 - 10.975) | 0.345 |  | 3.019 (0.520 - 17.520) | 0.218 |

*Abbreviations: mRS, modified Rankin Scale; OR; Odds Ratio; CV, average Constriction Velocity; MCV, Maximum Constriction Velocity; CH, percentage of constriction; DV, Dilation Velocity*

**Supplementary Table 11.** The results of the multivariable ordinal logistic regression for the prediction of a shift in the direction of a worse outcome on the 3-month mRS. All the AP parameters were adjusted for admission NIHSS, premorbid mRS, revascularization treatments, stroke volume, LVO, both DWM, and PVM Fazekas scores, hemorrhagic conversion, diabetes, and the concomitant use of β-blockers.

|  | 3-month mRS | |
| --- | --- | --- |
|  | **Common OR (95% confidence interval)** | **p-value** |
| CV mean | 0.822 (0.534 - 1.265) | 0.372 |
| CV homolateral | 0.825 (0.545 – 1.251) | 0.366 |
| CV contralateral | 0.850 (0.585 – 1.233) | 0.391 |
| MCV mean | 0.917 (0.691 - 1.217) | 0.549 |
| MCV contralateral | 0.940 (0.737 - 1.200) | 0.621 |
| CH contralateral | 1.000 (0.969 - 1.032) | 0.989 |
| DV contralateral | 0.827 (0.293 – 2.331) | 0.719 |
| DV mean | 0.445 (0.136 – 1.452) | 0.180 |

*Abbreviations: mRS, modified Rankin Scale; OR; Odds Ratio; CV, average Constriction Velocity; MCV, Maximum Constriction Velocity; CH, percentage of constriction; DV, Dilation Velocity*
